# De-novo headache with transient vertebro-basilar symptoms: role of embryonic hypoglossal artery

**DOI:** 10.1007/s10194-011-0394-5

**Published:** 2011-10-19

**Authors:** Angelo Maurizio Clerici, Giuseppe Craparo, Giuseppina Cafasso, Camilla Micieli, Giorgio Bono

**Affiliations:** 1 Neurology Unit, Ospedale di Circolo e Fondazione Macchi, University of Insubria, Viale Borri 57, 21100 Varese, Italy; 2 Neuroradiology Unit, Ospedale di Circolo e Fondazione Macchi, Viale Borri 57, 21100 Varese, Italy

**Keywords:** Persistent primitive hypoglossal artery, Acute headache, Carotid-basilar anastomosis, Transient ischemic attack, Vertebro-basilar system

## Abstract

We report the case of a 56-year-old man with acute onset of de-novo stabbing, pulsating and diffuse headache with subsequent appearance (within few minutes) of posterior fossa symptoms (vomiting, postural instability, anisocoria, incoordination, dysarthria, retropulsion) lasting 9–12 h. Recurrent hypertensive crises were detected during the acute observation in the Emergency Room, even in the absence of previous history of hypertension. Once subarachnoid hemorrhage and focal lesions (vascular and non-vascular) were excluded, brain computerized tomography-angiography and digital subtraction angiography disclosed the presence of left persistent primitive hypoglossal artery with bilateral vertebral artery hypoplasia and a slight aneurysmal dilation of the anterior communicating artery. Brain magnetic resonance study performed 24 h after onset of symptoms was negative for recent ischemic lesions. The clinical features of this rare vascular condition are discussed as a possible cause of magnetic resonance (diffusion weighted imaging) negative vertebro-basilar transient ischemic attack.

## Case report

A 56-year-old man was observed in the Emergency Room (ER) 40–50 min after the onset of acute de-novo stabbing, pulsating and diffuse headache with subsequent appearance (within few minutes) of dysarthria, postural instability with retropulsion, mild objective vertigo and vomiting, acral paresthesias of upper limbs, minimal confusional state without loss of consciousness or seizures. Neurological examination was relevant for reagent anisocoria (left smaller than right), slight speech disorder (dysarthria), retropulsion, moderate bilateral incoordination at the index-nose test, absence of fever and meningeal signs. Blood pressure (BP) was unstable with recurrent hypertensive crises (200/120 mmHg) during observation, in the absence of definite previous history of hypertension as well as of diabetes, headache, or cardiovascular problems. Electrocardiographic monitoring excluded paroxysmal arrhythmias, while cardiac enzymes and creatine-kinase were negative. In the ER, he was started on intravenous (i.v.) nimodipine (2 mg/hour) with progressive normalization of BP values within 4 h. Urgent brain computerized tomography (CT) was negative for hemorrhage and focal lesions.

Considering the unstable BP and the low NIHSS (National Institutes of Health Stroke Scale) score (=3) at presentation, i.v. thrombolysis for possible brainstem ischemia was excluded, and the patient was put on antiplatelet therapy with salicylic acid (i.v. 250 mg for 24 h, followed by oral administration) as for a minor ischemic events. Under i.v. salicylic acid, headache severity gradually decreased over the next 3 h, leaving only a slight diffuse, pulsating pain which remitted over the next 6 h. All the neurological signs and symptoms had completely recovered within 12 h.

**Fig. 1:**
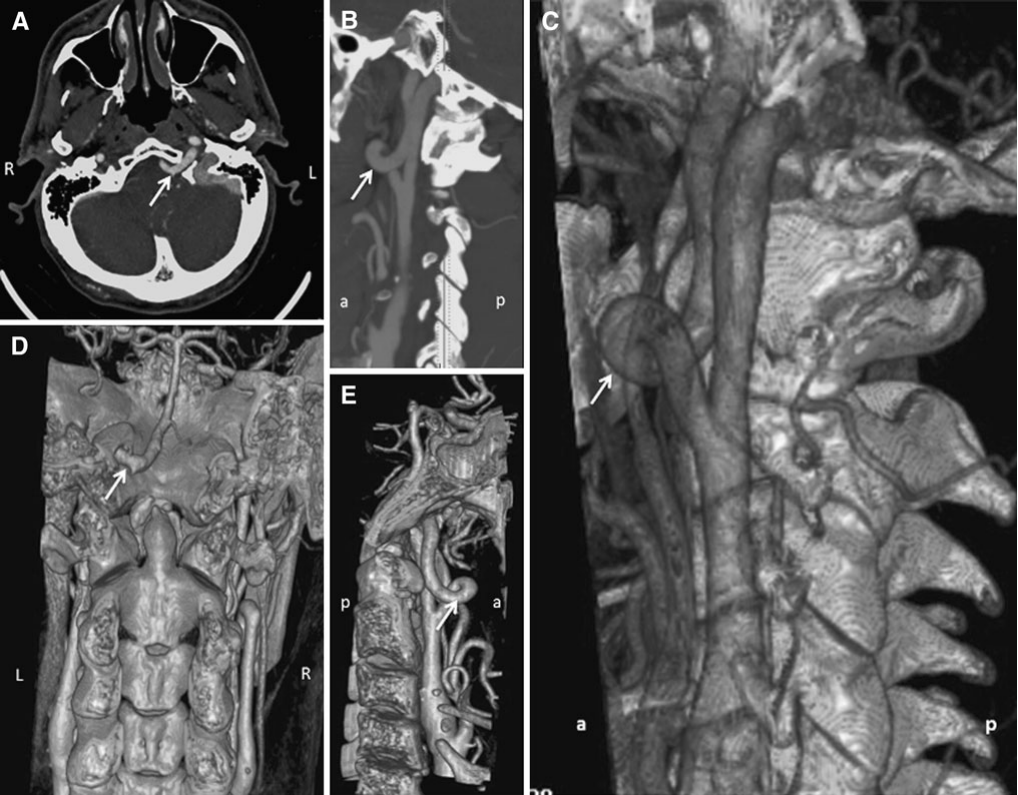
**a** Axial brain CT-angiography, **b**, **c** MIP and 3D-reconstruction CT-angiography showing left PPHA (*arrows*). **d** Three-dimensional-VR CT-angiography showing PPHA (*arrow*) entering the posterior cranial fossa through an enlarged hypoglossal canal and thus joining the lower portion of the basilar artery, and in **e** PPHA (*arrow*) as a large vessel originating from the internal carotid artery at the C2 vertebra level

In order to investigate the vertebro-basilar (VB) district, the patient also underwent urgent brain CT-angiography with maximum intensity projection (MIP), multiplanar (MP) and three-dimensional (3D) reconstructions, with the detection of left persistent primitive hypoglossal artery (PPHA) as a large vessel originating from the internal carotid artery (ICA) at the C2 vertebral level, entering the posterior cranial fossa through an enlarged hypoglossal canal and thus joining the lower portion of the basilar artery (Fig. 1). The study was then completed through 3D volume-rendering (VR) reconstructions for a better definition of the anatomical details (Fig. 1). Collateral findings were represented by bilateral vertebral artery hypoplasia together with a small saccular aneurysm (not-surgical) of the anterior communicating artery, confirmed by digital subtraction angiography (DSA) which revealed no additional vascular abnormalities. Brain magnetic resonance (MR) with MR-diffusion weighted imaging (MR-DWI) was performed 24 h after onset of symptoms and did not show recent ischemic lesions. The electroencephalogram—performed 12 h after admission—did not record epileptic/focal abnormalities. Uncommon causes of stroke (thrombophilia, patent foramen ovale and other relevant metabolic risk factors for stroke) were excluded during the course of the observation.

The patient was then discharged with ramipril (5 mg per day) plus oral salicylic acid (100 mg per day) and at the 6-month control no further episode of headache—with or without neurological symptoms—was reported.

## Comments

The clinical spectrum of acute “vascular” headache associated to VB signs and symptoms, once excluded critical conditions, could suggest at least four possible diagnoses: (1) basilar-type migraine, (2) posterior reversible encephalopathy syndrome (PRES), (3) headache attributed to benign (or reversible) angiopathy of the central nervous system, and (4) headache attributed to transient ischemic attack (TIA) or minor stroke, whose diagnostic criteria are summarized in Table 1.

**Table 1:** Diagnostic criteria for basilar-type migraine, posterior reversible encephalopathy syndrome (PRES), headache attributed to benign (or reversible) angiopathy of the central nervous system, and headache attributed to transient ischemic attack (TIA)

*Basilar-type migraine* [1]
Migraine with aura symptoms clearly originating from the brainstem and/or from both hemispheres simultaneously affected, but no motor weakness
(A) At least 2 attacks fulfilling criteria B–D
(B) Aura consisting of at least two of the following fully reversible symptoms, but no motor weakness: dysarthria, vertigo, tinnitus, hypacusia, diplopia, visual symptoms simultaneously in both temporal and nasal fields of both eyes, ataxia, decreased level of consciousness, simultaneously bilateral paraesthesias
(C) At least one of the following:
(1) At least one aura symptom develops gradually over ≥5 min and/or different aura symptoms occur in succession over ≥5 min
(2) Each aura symptom lasts ≥5 and ≤60 min
(D) Headache fulfilling criteria B–D for 1.1 *Migraine without aura* begins during the aura or follows aura within 60 min
(E) Not attributed to another disorder
*Posterior Reversible Encefalophathy Syndrome (PRES)* [2]
Acute or subacute neurologic presentation of headeache, nausea, vomiting, altered mental function, seizures, stupor, visual disturbances
Radiological hallmarks: reversible bilateral subcortical and cortical edema with a predominantly posterior distribution (parieto-occipital) at Fluid-Attenuated Inversion Recovery (FLAIR) MRI imaging
*Headache attributed to benign (or reversible) angiopathy of the central nervous system* [1]
(A) Diffuse, severe headache of abrupt or progressive onset, with or without focal neurological deficits and/or seizures and fulfilling criteria C and D
(B) “Strings and beads” appearance on angiography and subarachnoid hemorrhage ruled out by appropriate investigations
(C) One or both of the following:
(1) headache develops simultaneously with neurological deficits and/or seizures
(2) headache leads to angiography and discovery of “strings and beads” appearance
(D) Headache (and neurological deficits, if present) resolves spontaneously within 2 months
*Headache attributed to transient ischemic attack (TIA)* [1]
(A) Any new acute headache fulfilling criteria C and D
(B) Focal neurological deficit of ischemic origin lasting < 24 h
(C) Headache develops simultaneously with onset of focal deficit
(D) Headache resolves within 24 h

According to the current criteria of the International Classification of Headache Disorders—2nd Ed. (ICHD-II) [1], our case potentially fulfills only some of the diagnostic criteria for basilar-type migraine (“1.2.6”): headache, dysarthria, vertigo (with onset/worsening in few minutes), ataxia, decreased level of consciousness and simultaneous bilateral paraesthesias. However, the neurological symptoms have lasted over 60 min and were associated with de-novo headache (first episode), thus excluding the above hypothesis (Table 1).

The second diagnosis we have considered, is PRES, a rare and still poorly understood condition characterized by acute or subacute headache (usually thunderclap type), nausea, vomiting, altered mental function, seizures, stupor and/or visual disturbances, with the radiological hallmarks of reversible bilateral subcortical and cortical edema with a predominantly posterior distribution (parieto-occipital) at Fluid-Attenuated Inversion Recovery (FLAIR)-MR sequences [2].

PRES has been described in association with hypertensive encephalopathy, immunosuppressive and cytotoxic medications, puerperal eclampsia, collagen disease, renal failure, thrombotic thrombocytopenic purpura, human immunodeficiency virus infection, acute intermittent porphyria, and organ transplantation [2]. In our case, the lack of the typical MR findings and the negative electroencephalogram tend to rule out this diagnosis.

The third possible diagnosis is represented by “Headache attributed to benign (or reversible) angiopathy of the central nervous system” (ICHD-II “6.7.3”). In our case, despite the complete reversibility of all neurological signs, the lack of the radiological hallmarks “strings and beads” and the full headache recovery within 12 h, excludes this nosological entity (Table 1).

The last possible and more suitable diagnosis is that of “Headache attributed to transient ischemic attack (TIA)”—ICHD-II “6.1.2” (Table 1). In fact, headache occurs frequently in patients with acute cerebrovascular disorders with a frequency—according to different studies—ranging from 7 to 65% [3]. Several authors have stressed the greater incidence of headache among patients with ischemic events, particularly VB infarcts, due to the anatomical relationship between the posterior circulation and the trigeminal system [4]. Although relatively less frequent during TIA, sudden and acute headache of unknown cause, associated to dizziness or loss of balance, may represent a warning sign of VB failure. The most common symptoms of extracranial vertebral artery involvement are represented by dizziness, blurred vision and imbalance, while vertigo is more typical of intracranial vertebral artery disease. On the other hand, TIA due to basilar artery failure produces, more frequently, dizziness, double vision, dysphagia, slurred speech and unilateral/bilateral weakness [5].

Symptoms and signs due to TIA (either carotid or VB) must resolve—by definition—within 24 h but sometimes they may be very brief—lasting few minutes—probably due to sudden and temporary BP failure [5]. In this perspective, Sacco et al. have suggested to review the ICHD-II diagnostic criteria for “6.1.2” in the agreement with the new definition of TIA proposed by the American Heart Association Study Group. Accordingly, TIA is now defined as “a transient episode of neurological dysfunction caused by focal brain, spinal cord or retinal ischemia, without acute infarction” thus reducing the importance of the traditional temporal criterion for the definition of TIA (duration <24 h). As a consequence, brain-MR imaging becomes now the crucial recommended tool for TIA diagnosis [6].

Besides the above conditions discussed in the differential diagnosis versus the most probable hypothesis of “headache attributed to transient ischemic attack (TIA) or minor stroke”, we can also exclude ICHD-II “10.3.2” (Headache attributed to hypertensive crisis without hypertensive encephalopathy), due to the presence of long-lasting focal neurological signs.

In the present case, de-novo acute headache and transient ischemia in the posterior circulation are associated with the evidence of PPHA, a rare vascular anomaly of the posterior circulation that ensures a pathological anastomosis between carotid and basilar system.

At the intracranial level, four embryonic arteries (trigeminal, otic, hypoglossal and proatlantal) participate to the VB development: once completed, these vessels gradually regress, but in some cases they may persist [7]. The detection is often fortuitous during angiographic examination. The trigeminal artery is generally associated with disorders of the 3rd, 4th, 5th and 6th cranial nerves, while the otic artery may cause acoustic or facial nerves palsy [8].

The hypoglossal artery may lead to 12th nerve palsy, glossopharingeal neuralgia or vascular disorders in the posterior circulation. The estimated incidence of PPHA is 0.02–0.1% [7]. It originates as a branch of the cervical part of the ICA between C1 and C3 levels, passes through the hypoglossal canal and joins the lower portion of the basilar artery after a tortuous course. The vertebral arteries are usually hypoplastic and the ipsilateral to PPHA may be absent. Collateral vascular abnormalities are often represented by arteriovenous malformations or saccular aneurysms [7]. Consequently, any significant alteration of the blood flow in the proximal ICA may affect the posterior circulation with possible reversible VB symptoms. Conversely, thromboembolism from PPHA/carotid atherosclerotic plaques—less frequent—is usually associated to ischemic lesions [7].

From a pathogenetic point of view, it seems therefore reasonable to consider, in our case, the particular vulnerability of the posterior circulation due to the PPHA, the associated vascular abnormalities, and its possible secondary, transient, hemodynamic failure during hypertensive phases. Our clinical picture may be classified as a real TIA of the posterior circulation, also considering the complete clinical recovery within 24 h and the absence of ischemic lesions (including lacunar events) on MR investigations. Moreover, the headache presented by the patient, meets the ICHD-II diagnostic criteria for “Headache attributed to transient ischemic attack (TIA)”—subgroup “6.1.2”.

In conclusion, the persistence of carotid-basilar anastomoses, although rare entities, must be considered not only in patients with isolated or combined cranial nerve deficit, but also in the presence of cerebrovascular events involving the VB system. Their association with other vascular abnormalities (hypoplasia) can confer increased vulnerability to other factors (hypertension) as in the present case.
